# Supplementary material for: Glial Gap Junction Pathology in the Spinal Cord of the 5xFAD Mouse Model of Early-Onset Alzheimer’s Disease
Source: Int J Mol Sci. 2022 Dec 9;23(24):15597. doi: 10.3390/ijms232415597 (PMC9779687; doi:10.3390/ijms232415597)
Supplement: Supplementary file 1 [file ijms-23-15597-s001.zip › ijms-2036770-supplementary.pdf]

Supplementary Material

Supplementary Figures

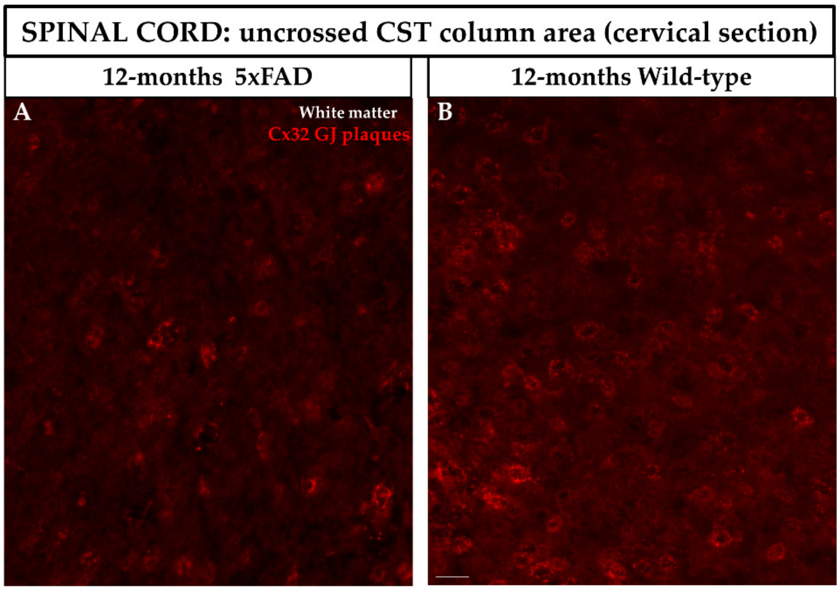

Supplementary Figure S1. Reduced number of Cx32-positive GJ plaques within the white matter of the 5xFAD spinal cord. Immunofluorescence staining was performed in cervical and lumbar (not shown) spinal cord sections from 12M 5xFAD (A) and WT (B) mice using an anti-Cx32 antibody (red). In 12M 5xFAD mice, the immunoreactive Cx32 GJ plaques appear reduced in the uncrossed corticospinal tract (CST) compared to their WT littermates. CST is a white matter column located at ventral funiculus and is responsible for the execution of movements and it innervates motor neurons of the proximal or axial musculature [1]. (A-B) Scale bars = 50 μm.

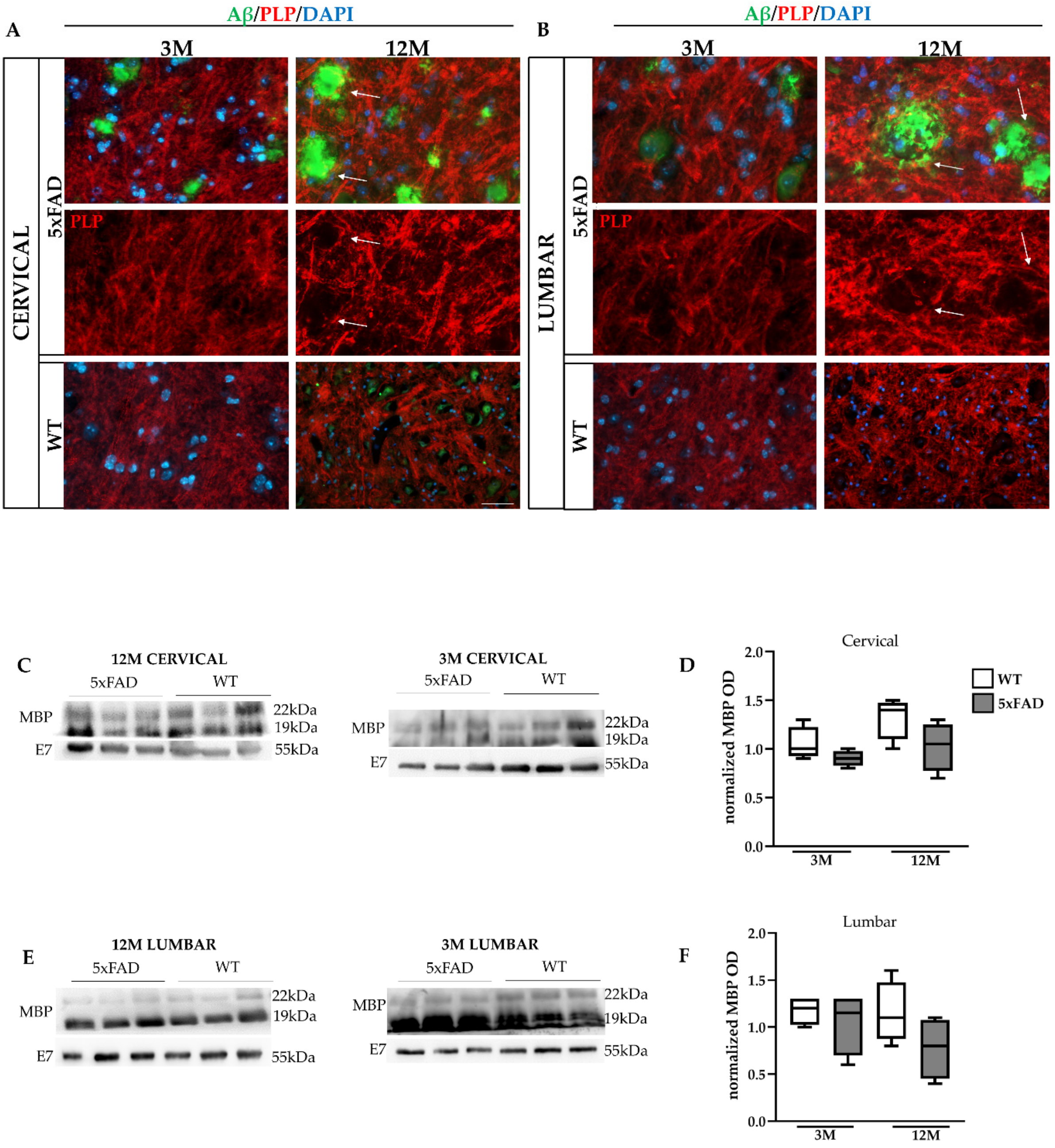

Supplementary Figure S2. Impaired myelination in the Aβ plaques microenvironment in the spinal cord of 5xFAD mice. (A-B) Double immunofluorescence staining of cervical and lumbar spinal cord GM from 5xFAD and WT mice with Aβ (green) and the myelin marker PLP (myelin proteolipid protein) (red). There is a disruption of the myelin microstructure focally in the area surrounding Aβ plaques (white arrows) in 12M 5xFAD mice compared to the WT group which shows a normal myelin distribution. (C-F) Immunoblot analysis and quantification of normalized band optic density (OD) revealed slightly lower but myelin basic protein (MBP) protein levels in 12M 5xFAD mice compared to the WT control group in both spinal cord levels but without reaching statistical significance. *β*-Tubulin (E7) was used as

loading control. The statistical analysis was performed by one-way ANOVA followed by Kruskal-Wallis multiple comparisons test (n = 6 in all groups). Data are presented as mean ± SD. (A-B) Scale bars = 50 μm; magnified view scale bar = 10 μm.

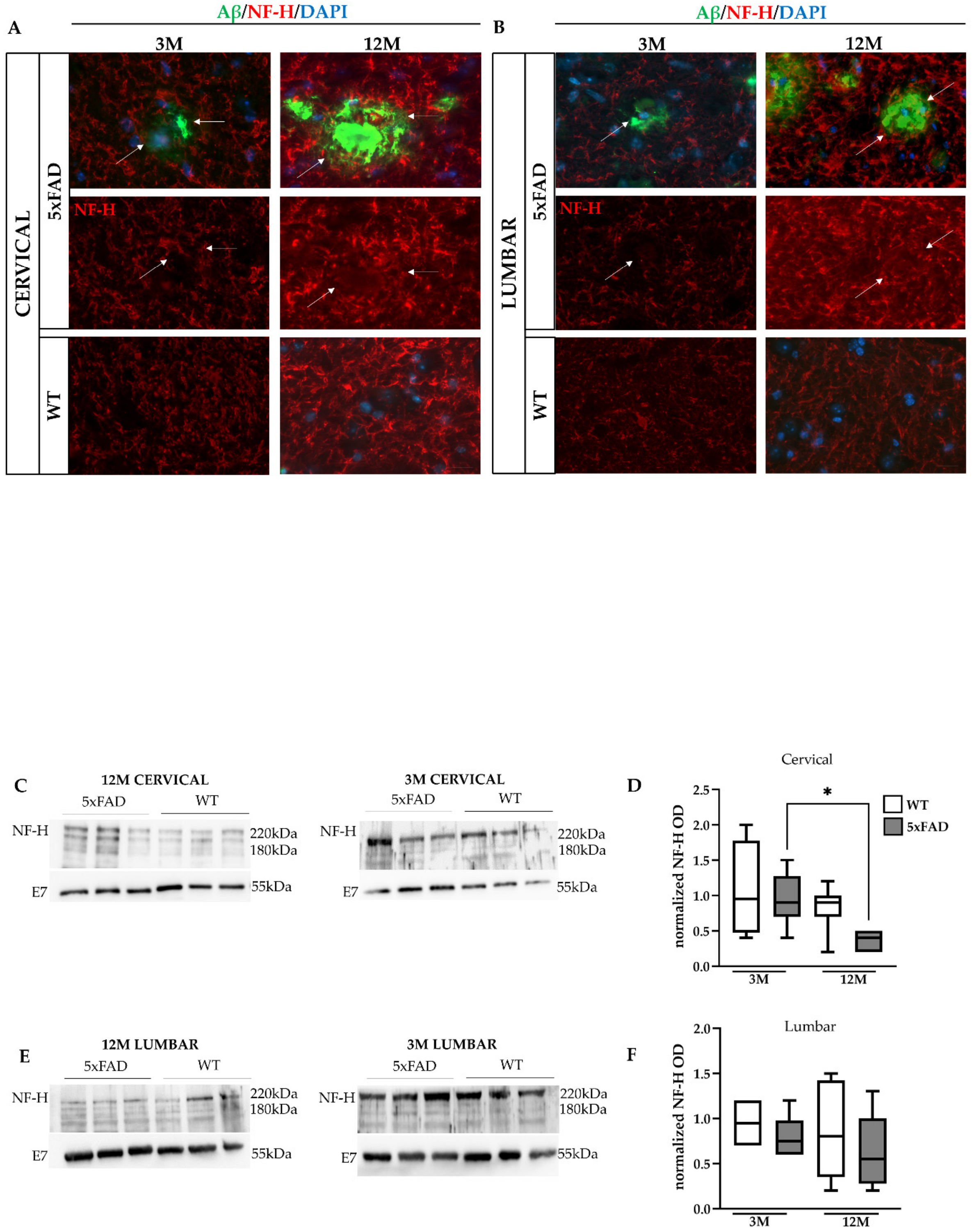

Supplementary Figure S3. Impaired axonal integrity in the Aβ plaques microenvironment in older 5xFAD mice. (A-B) Double immunofluorescence staining of cervical and lumbar spinal cord GM from 5xFAD and WT mice with Aβ (green) and the axonal marker NF-H (red). There is apparent disruption of NF-H immunoreactive axons indicated with white arrows in the Aβ plaques microenvironment in older 5xFAD mice. (C-F) Immunoblot analysis revealed overall similar NF-H levels in 12M 5xFAD mice compared to their WT littermates at both spinal cord

levels but the OD quantification showed a significant reduction in the cervical segment in 12- compared to 3-month-old 5xFAD mice. *β*-Tubulin (E7) was used as loading control. The statistical analysis of both immunoblots was performed by one-way ANOVA followed by Kruskal-Wallis multiple comparisons test (n = 6 in all groups). Data are presented as mean ± SD. (A-B) Scale bars = 50 μm; magnified view scale bar = 10 μm. Significance is given as: *p<0.05.
